# Leaders’ perspectives and actions to manage challenges in medical education presented by the COVID-19 pandemic: a nationwide survey of Japanese medical colleges

**DOI:** 10.1186/s12909-022-03193-1

**Published:** 2022-03-04

**Authors:** Mikio Hayashi, Takuya Saiki, Steven L. Kanter, Ming-Jung Ho

**Affiliations:** 1 Center for Medical Education, Kansai Medical University, Osaka, Japan; 2 Medical Education Development Center, Gifu University, Gifu, Japan; 3 Association of Academic Health Centers, Washington DC, USA; 4 Center for Innovation and Leadership in Education, Georgetown University Medical Center, 20057 Washington DC, USA

**Keywords:** COVID-19 pandemic, Leader’s perspectives, Nationwide survey, Thematic analysis, Sensemaking theory

## Abstract

**Background:**

The COVID-19 pandemic has caused medical colleges worldwide to suspend in-person classes and clinical clerkships. This fluid situation urgently required educators and learners to make a paradigm shift from traditional medical education. However, descriptions of how leaders manage policy decisions, especially considering cultural contexts, are limited. This study explores how the deans of medical colleges in Japan addressed the situations in which face-to-face contact is difficult and interacted with various stakeholders during the COVID-19 pandemic.

**Methods:**

The study employed a nationwide online survey by sending individual e-mails to the director of medical education at each of the 82 medical colleges in Japan. Responses were collected between May 26 and June 12, 2020 from the deans or directors of medical education. The survey questions were developed based on a literature review and consultations with international research collaborators. The survey asked what difficulties and opportunities were encountered through curriculum adjustments during the COVID-19 pandemic and what lessons could be shared with medical educators worldwide. Survey responses were analyzed using thematic analysis. The themes were categorized by stakeholder and then analyzed using the domains of sensemaking theory.

**Results:**

A total of 48 medical colleges in Japan completed the survey, yielding a response rate of 58.5%. The levels of participation in the study were 42.9%, 77.8%, and 74.2% among national, public, and private medical colleges, respectively, with responses from public and private medical colleges tending to be higher than those from national medical colleges. Japanese deans’ decisions for actions in adapting to COVID-19 involve perceiving cues from multiple stakeholder groups, including medical students, parents of medical students, medical faculties, and government officials. Thematic analysis of survey data reveals that Japanese deans’ actions in adapting to COVID-19 reflect characteristics of Japanese culture, with Japanese deans tending to emphasize in-depth introspection and collaboration with diverse stakeholders.

**Conclusions:**

Despite a lack of clear national guidelines for decision making, Japanese deans adapted to COVID-19 challenges by learning from one another and seeking the perspectives of a diverse group of stakeholders, aligned with local cultural context. Their approach offers important lessons for global medical educators.

**Supplementary Information:**

The online version contains supplementary material available at 10.1186/s12909-022-03193-1.

## Background

The COVID-19 pandemic has disrupted medical colleges worldwide from continuing with conventional training, such as in-person classes and clinical clerkships [1]. To cope with these unprecedented circumstances, institutions have provided online training [2, 3] and have explored alternative means to assess student performance [4]. This fluid situation urgently required educators and learners to engage in a paradigm shift from traditional medical training [5, 6]. The literature on adapting medical education to online formats during the pandemic has been growing rapidly [7, 8]. Medical education associations launched websites and offered webinars to share best practices and innovations during the pandemic [9, 10]. However, the majority of medical education research produced thus far regarding COVID-19 has focused on medical students and faculty with only limited discussion of how leaders handled policy recommendations and provided guidance across medical colleges [11]. A recent systematic review on the medical education adaptions to the pandemic noted that few papers discussed the process of adaptation [12]. This study explores the process of how leaders in Japanese medical colleges adapted to the pandemic using sensemaking theory, a theoretical framework widely used in the leadership literature to explain how to manage ongoing uncertainty[13]. Recent literature suggests to use it in the healthcare context for exploring challenges of adaptation [14]. The pandemic has created a level of ongoing uncertainty and presents a unique opportunity for a deeper understanding of sensemaking.

Japan, like a number of other countries, faced the challenges of the COVID-19 pandemic since January 2020. On April 16, 2020, a state of emergency was declared for all areas of Japan [15]. In the meantime, most educational institutions cancelled certain events at educational institutions, such as entrance and graduation ceremonies. Some medical colleges suspended clinical clerkships, and some implemented remote education for students. The general guidelines for COVID-19 countermeasures developed by the Japanese Ministry of Education, Culture, Sports, Science and Technology (MEXT) were sent to all higher education institutions, including medical colleges, on June 5, 2020 [16]. These guidelines for higher education in Japan were not developed specifically for medical colleges. In the US, the Association of American Medical Colleges issued guidance for medical colleges with regular updates [17]. In the UK, the General Medical Council and Medical Schools Council worked together on various statements for medical colleges [18]. In Japan, conversely, the main advice given was that colleges should operate in a resilient manner based on the guidelines, which were general in nature, not addressing the special nature of medicine and its experiential educational practices such as clinical clerkships, anatomy practice, and objective structured clinical examinations. The situation in Japan required medical college leaders to sense potentially problematic issues, make interpretations, and take action within the local contexts. Each Japanese medical college had to consider the content of medical education according to the extent of the pandemic in each region in which the college was located and the regulations of the local government. Furthermore, the Japanese deans have to take actions according to Japanese cultural values on collectivism, power hierarchy, and uncertainty avoidance [19–21]. Considering cultural differences in medical education [22–24], examining how COVID-19 is managed in different cultural contexts offers important lessons for global medical educators to plan for the future.

## Method

### Setting

Japan has a total of 82 medical colleges, divided into six main geographic regions: Tohoku and Hokkaido; Kanto; Tokai and Hokuriku; Kinki; Chugoku and Shikoku; and Kyushu. Specifically, 42 are national, 8 are public, 31 are private, and 1 is a national defence medical college. There is a preexisting electronic mailing list, named Association of the Departments of Medical Education, for the purpose of exchanging information among Japanese medical colleges. This mailing list was an important resource for medical colleges at the time of the pandemic [25, 26]. All medical colleges offer 6-year programs with a capacity of 80–120 students per year. Clinical clerkships typically are conducted in the fourth, fifth, and sixth years of study. Medical students are required to complete two years of post-graduate clinical training after graduating and passing the national examination [27].

### Study design

The authors followed the Standards for Reporting Qualitative Research (SRQR) recommendations [28]. The authors developed questions based on a review of the relevant literature [7, 8], and consultations with international research collaborators in Italy, Korea, Taiwan, and the United States, where similar surveys have been conducted (Appendix 1) [29–31]. The study documented the challenges and adaptations that medical education deans critically considered to continue medical education amid the pandemic by surveying the deans of medical colleges in Japan on the following topics:

1. What curricular adjustments have been made to offer medical education during the COVID-19 pandemic?
2. What were the major challenges to medical education due to COVID-19?
3. What are the opportunities for medical education reform due to COVID-19?
4. What were lessons learned from responding to COVID-19 that are particularly worth sharing with medical educators worldwide?

### Data collection

The study employed an online survey, which was distributed to 82 medical colleges in Japan. The first author (MH) sent individual e-mails to the director of medical education at each medical college, requesting that an online survey by completed by the dean. Responses were collected from the deans of medical colleges who are responsible for overseeing all missions of their colleges, including education, research, clinical care, and community engagement. If the dean was unavailable, the director of medical education, who is responsible for overseeing the educational activities of the medical college was invited to complete the survey. To encourage participation, we sent, via e-mail, invitations containing information about the purpose of the study, data confidentiality, and a weblink to the online survey. Participation was voluntary without incentives offered. No personal contact was made with the deans. Following the initial e-mail, we periodically sent reminders. Responses were collected between May 26 and June 12, 2020. The online written surveys were conducted in Japanese, and the responses were collected in Japanese. After collecting the responses in Japanese, the first author (MH) translated all the responses into English for the international research team to analyze. These contents were checked by the second author (TS) to ensure the accuracy of the translation.

### Ethics

The study protocol was developed in accordance to the ethical guideline at Georgetown University. This study was deemed exempt by the Institutional Review Board of Georgetown University. The participants were informed of the study’s scope and nature, and informed consent was obtained from all of them.

### Data analysis

Free-text responses to the open-ended questions in the survey were analyzed using thematic analysis, which consists of generative coding and theorizing to identify instances of similar concepts in the data set [32, 33]. Then we analyzed the themes using the framework of sensemaking theory [13].

Sensemaking theory is an important theory, widely used in education and healthcare research to interpret organizational behavior [34, 35]. A sensemaking process is triggered when an individual confronts issues that are confusing, and consists of three domains: perceiving cues, creating interpretations, and taking actions [14]. When an individual encounters a moment of ambiguity or uncertainty, they seek to clarify what is going on by extracting and interpreting cues from their environment, and then they use those interpretations to determine how to act on the environment. After the themes emerged, the authors grouped them into two categories: perceiving cues and taking actions.

The first author (MH) was formally trained in NVivo11 for Windows (QSR International, Australia; a program that supports analysis of qualitative data). MH conducted all steps of coding, including reading and rereading of free-text responses until themes were identified. Then themes were categorized into main and subcategories and tabulated using NVivo11 to identify their frequency. The themes extracted were categorized by stakeholder groups and then analyzed using the domains of sensemaking theory. The first author (MH) identified the themes that emerged from qualitative data; the second (TS) and senior (MJH) authors checked the analysis. For each point of disagreement, the three researchers discussed and reviewed the data until a consensus was reached. Our research team included two Japanese medical educators (MH, TS) and two American medical educators (SK, MJH). While the Japanese researchers bring the emic perspectives and associated richness and potential bias, the American researchers balance the emic view with the etic perspective. Furthermore, the research team has experience in leadership practice (TS and SK) as well as expertise in leadership scholarship (MH and MJH).

## Results

A total of 48 of 82 medical colleges in Japan completed the survey (a response rate of 58.5%). Table 1 provides characteristics of the medical colleges. The levels of participation in the study were 42.9%, 77.8%, and 74.2% among national, public, and private medical colleges, respectively, with responses from public and private medical colleges tending to be higher than those from national medical colleges. Data revealed that deans faced a variety of challenges and, in the absence of clear guidelines, were making their own interpretations and taking actions. Thematic analysis of survey data reveals the involvement of multiple stakeholder groups, including medical students, parents of medical students, medical faculties, and government. Table 2 presents the themes and representative quotes. The authors did not observe any overt difference in the responses among the geographical regions.

**Table 1:** Characteristics of medical colleges

Characteristic	No. Respondents (No. All Japanese Medical Colleges) (*n*)	Response rate (%)
**Ownership**		
National	18 (42)	42.9
Public	7 (9)	77.8
Private	23 (31)	74.2
**Area**		
Tohoku and Hokkaido regions	6 (10)	60.0
Kanto region	19 (28)	67.9
Tokai and Hokuriku regions	6 (11)	54.5
Kinki region	6 (12)	50.0
Chugoku and Shikoku regions	5 (10)	50.0
Kyushu region	6 (11)	54.5
**Total**	48 (82)	58.5

Japan has a total of 82 medical colleges, divided into six main geographic regions: Tohoku and Hokkaido; Kanto; Tokai and Hokuriku; Kinki; Chugoku and Shikoku; and Kyushu. A total of 48 of 82 medical colleges in Japan completed the survey (a response rate of 58.5%)

**Table 2:** Themes and quotes organized by stakeholder group

Themes/ Stakeholders	Subthemes/ Quotes
Perceiving Cues/ Medical Students	**Changes in educational approach** “Clinical clerkship is postponed during periods when the hospital is implementing patient restrictions and other measures. Even after clinical clerkship begins, direct patient contact training is not allowed during the period when COVID-19 is not settled. The curriculum was forced to be mainly observation-based.” **Professional Development** “I am concerned the lack of group activities and behaviors in the lower grades will lead to inadequate development of professionalism as a member of society or as a person rather than as a doctor.” **Increased financial burden** “As a private medical college, the high cost of tuition is a burden on students. We need to support students who are losing their part-time jobs and income.”
Perceiving Cues/ Parents	**Criticisms from parents** “Parents complained at a time when a state of emergency was being declared. In addition, clinical clerkship started just before the end of the emergency declaration.” **Concerns about code of conduct** “Students are expected to comply with the medical college’s code of conduct, but there is no way to ensure their certainty and safety.” **Perspective change about medical profession** “Although medical professionals have been required by society to make a certain amount of self-sacrifice, from now on, the importance of managing and maintaining one’s physical and mental health will be emphasized as a professional. The content of altruism may have to be reviewed.”
Perceiving Cues/ Medical Faculties	**Excessive workload due to the limited budget and manpower** “There is the issue of increasing the workload of the administrative staff due to the change in class format and securing a temporary budget for equipment preparation and other expenses.” **Rapid transition to online educational environment** “I believe that the paradigm shift stress regarding the way interpersonal relationships are built online and online from the era of face to face.” **Awareness of cooperation with other medical colleges** “I realized the importance of cooperation between colleges.” “The exchange of information by medical educators in the country was particularly important.”
Perceiving Cues/ Government	**Limited clinical clerkships associated with infection control** “All clinical clerkships using off-campus facilities were cancelled. As a medical college, we have no experience at all with risk assessment and infection prevention measures for students resuming classes. There is a tendency to excessively restrict students’ participation in hospital training.” **Fluid policy changes** “The situation is changing so rapidly that the government’s instructions are being delayed. It is difficult to understand the government’s instructions and decisions regarding the handling of clinical clerkship at a distant environment and the determination of advancement and graduation.”
Taking Actions/ Medical Students	**Revising the curriculum in response to the online** “The introduction of e-learning, which has been an educational challenge, should be accomplished within a specified period of time. We will consolidate the educational content that had to be revised as a result of this situation, including the clarification of educational goals.” **Attentive online support** “The medical college mailed the necessary documents to each student to prepare for the distance learning course, which the students used to prepare for the course. The advisors assisted students and made sure that all students were ready to take the course before the start of classes.” **Creating alternative learning task** “In case of absence due to illness, all face-to-face lectures are recorded to avoid disadvantages in terms of learning. Students can watch the lectures on demand. Even if a student is absent from a clinical clerkship, he or she is given an assignment and asked to submit a report for evaluation.” **Setting up a working group with medical students** “A public relation’s working group, in which students also participate, has been set up to examine how to accurately get information from the medical college to all students.”
Taking Actions/ Parents	**Communication of information** “A new email newsletter has been issued to share individual student inquiries with the entire faculty and to promptly notify decisions made by the Academic Affairs Committee and other meetings. By the end of May, 13 issues of the magazine had been published, and almost all students had subscribed to it. It is helping to dispel anxiety in an environment that makes it impossible to go to medical college.” **Thoughtful response through enhanced cooperation** “We are cooperating each other to gather feedback from students about learning issues due to the continued presence of distance learning, and concerns of students and their families about the start of college to address those concerns appropriately. In preparation for the resumption of classes, an educational video on COVID-19 will be made for viewing by students as well as parents to encourage them to act with the right knowledge.”
Taking Actions/ Medical Faculties	**Provision of education to faculties** “It is important to educate students about information literacy, but I also feel the need for faculty development to adapt to these changes.” **Efforts to reduce the burden on faculties** “Digital education, which has been a major issue for Japan up to now, is now being promoted across the board. From now on, we would like to develop more effective education through the effective use of digital education in combination.” **Exploring educational policy through collaboration** “I think it is important to make quick decisions and take action. In Japan, information sharing among medical colleges is also done quickly, which was very helpful. I don’t know how it is in other countries, but I think the network of medical educators in Japan is functioning well.”
Taking Actions/ Government	**Ensuring safety** “The week of medical college attendance is determined by infection grade level. Check yourself for fever, wear a mask, and use a large room to avoid crowding. Also open the windows for ventilation.” **Expanding Options** “As a general rule, clinical clerkship is conducted only at medical college hospitals, and preparations are made so that clinical clerkship can be done while switching between on-site and off-site training, depending on the prevalence of the disease.”

### General process of Japanese curricular adaptations

Japanese deans reported that they had steered online education according to their universities’ learning environments and the national government’s declaration of a state of emergency. The deans mentioned that during this process, they also adopted other universities’ policy changes and referenced discussions from the Association of Departments of Medical Education as a guide for changing policy at their individual universities. Before making policy changes, they paid close attention to many inquiries, not only from medical students and faculty members but also from medical students’ parents. Finally, the deans emphasized the importance of aligning policy with that of neighboring medical universities.

Although the pandemic required quick decisions, especially in the beginning, the Japanese deans did not act too hastily and adopted a trial-and-error approach. They first integrated various stakeholders’ opinions and examined various possibilities. Rather than simply changing policies, the deans sought ways to support their medical students. During this process, the deans deeply considered how, as leaders, they could also ease teachers’ burdens and parents’ concerns.

### Perceiving cues

Some themes, identified during thematic analysis, revealed the cues that deans sensed in their environments, and then recognized as important and relevant to dealing effectively with the pandemic. We present these themes below, categorized by stakeholder.

#### Medical students

The deans recognized that medical students had to adjust to clinical clerkship with less direct contact with patients to abide by infection control measures in hospitals. In addition to a compromised clinical experience, deans expressed concerns about the development of medical students as professionals and about their transition from undergraduate education to post-graduate clinical training. Regarding the trend to adjust standards on the duration and content of clinical clerkships to avoid postponement of promotion or graduation, the respondents expressed concern that such changes might lead to insufficient competencies. Other concerns about students noted were the financial burden of medical students and related issues.

#### Parents

The respondents raised the challenge of dealing with numerous criticisms from parents of medical students about the restart of classes and clinical clerkships, especially in geographic areas where COVID-19 was more prevalent. In such areas, parents complained that clinical clerkships had started just before the end of the emergency declaration. Although many medical colleges had established strategies regarding codes of conduct, such as stay-at-home policies, parents continued to be critical of their effectiveness in ensuring the safety of their children. The deans were also concerned about media coverage of the pandemic and how it might influence the perceptions of medical students and their parents regarding the value of the medical profession.

#### Medical faculties

The deans were challenged by their faculties potentially facing excessive workloads as a result of COVID-19. Furthermore, they were concerned about the potential exhaustion and stress of faculty members working in clinical areas. Deans expressed hesitation about whether faculty members would be able to adequately adapt to the process of rapid transition to an online educational environment. Specifically, the deans were struggling to allocate budget and personnel to address the changes in the environment caused by COVID-19. They recognized the difficulties in creating an adequate educational environment under the current circumstances.

#### Government

There were numerous considerations of government policy in response to the pandemic. The government advised the universities to operate in a resilient manner without consideration of the special features of medical education emphasizing experiential learning in clinical settings. Leaders of medical colleges are entrusted to operationalize the general guidelines at their discretions. In the survey, the deans expressed concerns about excessive restriction of learning opportunities that may be required by infection control measures. Deans also acknowledged the frustrations voiced by faculty members in terms of having to make fluid policy changes.

### Taking actions

This section demonstrates the role of action in sensemaking. Themes related to what the deans are doing within their medical colleges in the context of the pandemic will be presented by stakeholder category.

#### Medical students

The medical colleges reviewed their educational objectives for lectures and clinical clerkships in response to the trend to move the learning environment online. The deans tasked advisors with providing detailed individualized attention to prepare students for classes, in addition to using manuals in the transition to online courses. If students were unable to participate in a class or clerkship due to illness, they could study later. The deans and faculty members, in collaboration with medical students, set up a working group to solicit the opinion of medical students on educational policies. They aimed to prevent students from exclusion from this cooperative approach. Through the above process, both the faculty members who reviewed the curriculum and the medical students themselves discussed what competencies should be acquired throughout their whole curriculum.

#### Parents

Each medical college devised means, through electronic media, to communicate information to students and parents and ensure thorough information dissemination and accountability. A new committee was formed within the medical college to review the responses from students and parents. With the prolonged period of distance learning, deans aimed to take into consideration the concerns of the students as well as their parents, and the new committee was an essential part of this process, especially in responding appropriately to various complaints from parents.

#### Medical faculties

The deans were mindful of the need for faculty development in the process of the rapid transition of the educational environment to online. There was a trend to search for ways to reduce the burden on faculty while developing effective online education modules. Medical college deans relied on the cooperation of other medical college deans as they were developing new educational policies for implementation at their own medical colleges. The deans used a trial-and-error approach, first implementing something new, and then making corrections based on feedback and lessons learned. Deans also reminded the faculty and supervisors that they are role models for medical students and so their actions matter.

#### Government

The deans ensured the importance of infection control, and based on their own consensus, decided on students’ personal protective equipment options, attendance standards, and educational choices. They planned what to do if ongoing clinical clerkships had to be interrupted due to local infection rates, and each had several options regarding learning methods. Deans respected the discretionary authority of faculty members in teaching clinical clerkships, and solicited the opinions of faculty members in each department to determine responses to the general government policies.

## Discussion

Based on thematic analysis, and using a lens of sensemaking theory, we revealed how Japanese medical college deans perceived cues from various stakeholders and then took action to meet institutional and social challenges at the beginning of the COVID-19 pandemic. In the absence of clear governmental guidelines for medical colleges, this study illuminates how college policies were enacted, taking into account specific local situations at an institution, as well as those encountered at other medical colleges. Although sensemaking theory cannot elucidate how diverse stakeholders make sense and react to a crisis simultaneously [14], this theory is appropriate for this study in helping to clarify how deans understand and react to the situation during the pandemic. The process of the deans learning from each other and seeking collaboration with diverse stakeholders is an authentic example of effective leadership during a crisis and offers an important lesson for global medical educators because it is clear evidence of leadership principles in action [36].

Most of the results of this study provide additional support for the findings of extant medical education literature on adaptation during the COVID-19 pandemic, which highlight medical students’ adaptation to teaching, learning, and support aspects, and the need for stakeholder-specific responses [29, 37, 38]. Furthermore, this study addressed the following gaps in literature: 1. Previous studies have pointed out that integrated data, rather than data from a single institution, is needed to examine the adaptation process [39]; 2. Previous studies called for analysis of organizational programs and levels in a variety of ways [29]; 3. Previous studies have not acknowledged the importance of the responses of parents and the fact that Japanese deans can influence the sensemaking process in a variety of ways. Although it has been shown that the adaptation of medical students to the COVID-19 pandemic must be carefully considered because of their vulnerable position in their respective institutions [40, 41], the results of this study indicate that their parents can influence the decisions of Japanese deans. The authors also believe that the results may inform medical educators in other countries when exploring the impact on the student–teacher relationship. Although it was difficult to make inter-regional comparisons within Japan based on the themes extracted in this study, a comparison with a similar study in Italy revealed the influence of strong government guidance on the decision-making process in Japan [29]. In other words, in Italy, strong government guidance led to collaboration among those in charge, while in Japan, similar collaboration was apparent despite the absence of any guideline from the government. Although the importance of collaboration has been suggested in previous studies [1, 29], the authors believe that exploring the factors involved in inter-regional collaboration, based on this study, may be a future research topic.

Sensemaking theory is generally regarded as a social theory because even individuals making sense on their own are embedded in a social context [13, 14]. The interpretation of Japanese medical deans to cues during the pandemic may be due, in part, to Japanese cultural characteristics, including “power distance” and uncertainty avoidance. Hofstede defines “power distance” as “the extent to which the less powerful members of institutions and organizations within a country expect and accept that power is distributed unequally” [19]. Hofstede notes that, in societies with large power disparities, it is common for policy decisions to be centralized and that there is a tendency for the relationships between subordinates and supervisors to be emotional rather than practical [20]. Even though the pandemic required quick decisions, approaches to these situations were consistent with what the Japanese deans perceived as cues from various stakeholders, along with in-depth introspection (*hansei*) [42].

Hofstede also observes that Japanese culture tends to be more collectivist than individualist, and that collectivism tends to be exclusionary and considers social networks as its main source of information. Furthermore, there is a basic aim to achieve social harmony and consensus rather than self-fulfillment. Collectivism tends to prioritize the interests of the group over those of the individual[21]. Japanese cultural characteristics also are found in the domain of taking action, which is similar to another study that showed that cultural characteristics influenced decisions and actions of Taiwanese medical students when faced with professionalism dilemmas [43]. In our survey results, the deans reported that many faculty members were too busy to reflect on online education, and so deans promoted collaboration with other medical colleges to overcome resistance to the rapid transition to online education. As illustrated by the experiences of Japanese deans, decision making that is aligned with cultural contexts is important for determining effective action.

Although this study has shown that Japanese deans have learned many lessons from other deans, sought cooperation and input from diverse stakeholders, and taken various actions based on others’ perspectives by aligning their intentions with cultural values, there are some limitations to be considered. First, while sensemaking theory provides a useful lens to understand the responses of Japanese medical education leaders, the process of discerning interpretations is beyond the scope of this study. Further research, using in-depth interview methods, might illuminate the process of interpretation of the decision makers. Second, we identified stakeholders involved in the Japanese medical colleges’ adaptations to COVID-19; the organizations where the study participants work are complex and dynamic social environments [44]. Furthermore, this study did not fully capture the details of the concerns of the deans regarding the professional development of medical students and the individual responses to those concerns. Further investigation of the complex interactions among the stakeholders would require in-depth interviews with medical faculties and medical students as well as the deans of the medical colleges. Another limitation is that it lacks a long-term perspective. Continuous research regarding the challenges and adaptations in medical education would be required as the status of COVID-19 pandemic is rapidly changing. Finally, the response rate of this online survey was 58.5%, and the fact that the percentage of national medical colleges participating in this study was smaller than that of public and private medical colleges should be considered a limitation of this study.

## Conclusions

This study explores how the deans of medical colleges in Japan addressed the challenges caused by the COVID-19 pandemic, including incorporation of stakeholder concerns. Deans faced the task of managing challenges in the absence of clear national guidelines for medical colleges, and thus relied on learning from other deans and seeking collaboration and input from diverse stakeholders, while aligning decisions with cultural values. These findings offer important lessons for global medical educators to consider learner-centered approaches and perspectives of various stakeholders in local contexts when planning for future strategies to cope with pandemics and other crises.

## Supplementary Information

**Additional file 1: Supplemental Digital Appendix 1.** Survey Questions.

## Data Availability

The datasets generated and/or analysed during the current study are not publicly available due to protect the originality of our work so that we can continue evaluating future examinees, but are available from the corresponding author on reasonable request.
