# Supplementary material for: Leaders’ perspectives and actions to manage challenges in medical education presented by the COVID-19 pandemic: a nationwide survey of Japanese medical colleges
Source: BMC Med Educ. 2022 Mar 4;22:144. doi: 10.1186/s12909-022-03193-1 (PMC8894829; doi:10.1186/s12909-022-03193-1)
Supplement: Supplementary file 1 — Additional file 1: Supplemental Digital Appendix 1. Survey Questions. [file 12909_2022_3193_MOESM1_ESM.docx]

**Supplemental Digital Appendix 1. Survey Questions**

1. How are you adjusting your medical school curriculum in response to the COVID-19 pandemic?

1.1. What are the adaptations in clinical curriculum?

1.2. What are the adaptations in basic science curriculum?

1.3. What are the adaptations in student evaluation?

1.4. What are the adaptations in student counseling?

2. What are the major challenges to your medical school due to the COVID-19 pandemic?

2.1. What are the challenges from students (e.g. not following social distancing guidelines), parents (e.g. requesting to stop clinical activities), patients (e.g. refusing to be seen by students), and government (e.g. issuing guidelines to stop gatherings of more than 10 people) in response to COVID-19?

2.2. Did the COVID-19 pandemic bring special challenges to professionalism and ethics education?

3. What are the opportunities for medical education reform arising from the COVID-19 pandemic?

3.1. Which curriculum adaptations would be continued after the pandemic is contained?

3.2. Has this pandemic changed your opinions about medical education, such as strengths/weaknesses of previous approaches, future trends?

4. Have previous pandemics, such as SARS or AIDS, impacted medical education in your country?

5. What are the lessons you want to share with medical educators around the world regarding how your medical school responded to the COVID-19 pandemic?

6. Regarding the impact of COVID-19 on medical education, do you have other thoughts to share?
